# Judicial Homicide

**Published:** 1846-03-01

**Authors:** 


*Judicial Homicide.*—The case of Dr. Baker, who was some time since executed in Kentucky, for the murder of his brother-in-law, (of which our readers have probably seen some accounts in the newspapers,) appears to have been of a character to give occasion for the expression which we have prefixed to this article. If the facts which we find presented in the January No. of the western Journal of Medicine, by Dr. Yandell, one of the editors of the Journal, exhibit a correct view of the case, (and we see no reason to doubt it,) the execution was neither more nor less than *the deliberate murder of a monomaniac agreeably to the sentence of the law!* The circumstances as detailed in the account just referred to, were briefly these: The unfortunate victim, who occupied a reputable position as a medical practitioner, became possessed with the most extraordinary ideas of the unchastity of his wife. He asserted to his father-in-law, and others, that she was seduced when only nine years of age by her teacher, a clergyman whose moral character was unimpeached; that his brother-in-law, whom he afterwards killed, had come to his bed in the night and communicated with her in his presence, that she had had criminal intercourse with several of her uncles, as also with various others, amongst whom was a negro servant. These charges he made freely to various persons. He accused his step-mother and his sisters of lewdness. Bates, the brother-in-law, he believed intended to kill him, and he appears to have labored under a constant fear of assassination by him or his emissaries. Under the influence of these delusions he deliberately shot him with a pistol, after which he went to the home of an uncle, stated what he done, appeared highly elated, and expressed no fears if he could get justice.

The plea of mental aberration, which his counsel set up in his defence, was not with his consent: On the other hand, he declared he would "rather be hung than that such a plea should be relied on." He wished to plead justification; that Bates was at the head of a conspiracy to take his life, and he could prove it by Mrs. Bates. He maintained the same asseverations with vehemence under the gallows, protesting to the last his wife's infamy, and his own justification.

Various collateral circumstances were adduced on the trial to substantiate his insanity, such as his peculiar restless and violent deportment, his suspicious and irritable temperament, extraordinary conduct on various occasions, watchfulness, coldness of extremities, costiveness, irritability of stomach, &c. On the other hand, not a shadow of evidence was elicited that his wife was guilty of any of the charges.

In view of these facts can there be a question that there existed a "*reasonable doubt*" whether the act was committed under a rational sense of responsibility? Three medical men,—whom we infer from the account were the only physicians examined—testified to the existence of monomania. One of these was the late Prof. Richardson, of Transylvania University. The verdict however, was "guilty of wilful murder," and the sentence was pronounced. Considerable effort was afterward made to induce the Governor to pardon him. The physicians of Lexington, Frankfort, Danville, Lancaster and Louisville united in communicating to the Governor their solemn conviction of his insanity. It all availed nothing, and the sentence was executed.

For the honor of science, justice and humanity, it is to be hoped another instance of such seeming ignorance and barbarity will not soon disgrace the legal tribunals of our country. The jury, it is stated, could not understand that a man could be sane on one subject, while on others he evinced a sound understanding. Nor does this fact appear to have been recognized by the Judge. One of his questions to a medical witness was "whether such a disease as *monomania* was known and recognized in medical authority." On being answered that it was, both in law and medicine, he replied, in the hearing of the jury, "that he only made the inquiry as to medicine—he claimed to know something of the law himself." Since, if it were recognized in law it would be presumed to be so in medicine, it is fair to infer from this language that he did not regard it a valid legal distinction.

That the Governor of the State should not interpose under the circumstances, and in view of the extent and unanimity of medical opinion on the subject, is almost incredible. It is a melancholy instance of a want of confidence in the profession. An unfortunate fellow being, who, by a jury of impartial, intelligent physicians would have been recommended as a candidate for an insane asylum, afflicted with a disorder which of all others should excite sympathy, and secure protection and care, and who, it is probable, by proper management, might have been restored to himself, his family and society, expiates the commission of an insane act on the gallows! The reader cannot but exclaim—thank God I am spared the remorse which should be felt by all the official actors in such a tragedy!

